# A Personal Journey Through the Microbial World in Quilting

**DOI:** 10.3201/eid3110.AC3110

**Published:** 2025-10

**Authors:** Lesli Mitchell

**Affiliations:** Centers for Disease Control and Prevention, Atlanta, Georgia, USA

**Keywords:** Mija-tesse Ververs, The Pathogen Quilt, quilting, microbial, art-science connection, Vibrio cholerae, Ebola, HIV/AIDS, SARS-CoV-2, COVID-19 influenza, Nipah virus, monkeypox virus

**Figure:**
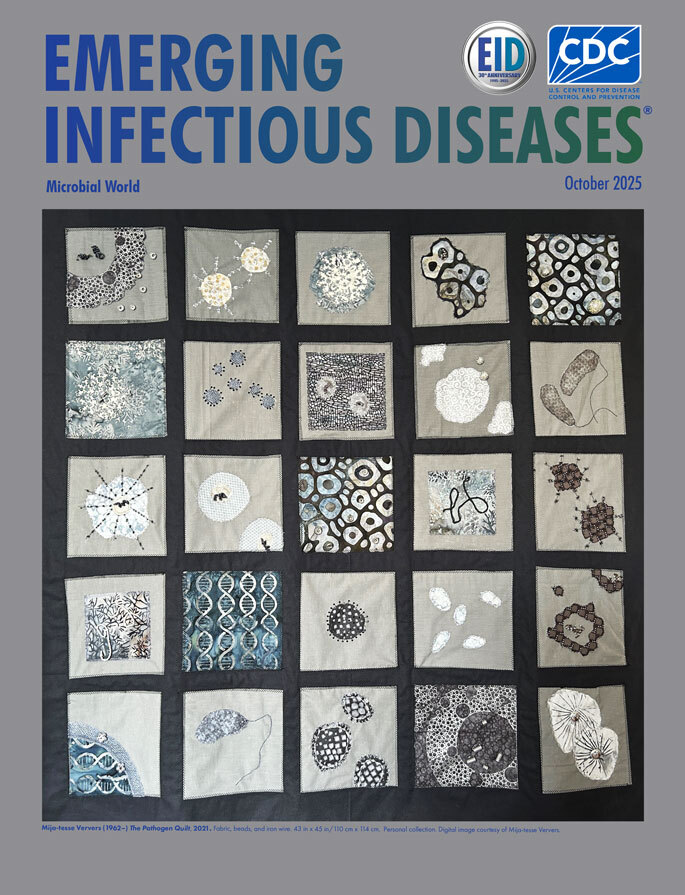
**Mija-tesse Ververs (1962–), *The Pathogen Quilt*, 2021**. Fabric, beads, and iron wire. 43 in x 45 in/110 cm x 114 cm. Personal collection. Digital image courtesy of Mija-tesse Ververs.

This month’s cover features *The Pathogen Quilt*, created by Mija-tesse Ververs, MMed, MPH, a health scientist at the Centers for Disease Control and Prevention. The quilt created by the artist serves as a poignant reflection on global health challenges, particularly in the wake of the COVID-19 pandemic. It highlights a variety of pathogens from the microbial world, including *Vibrio cholerae*, Ebola virus, HIV, SARS-CoV-2, influenza virus, Nipah virus, and mpox virus (Figure). Each pathogen is represented not only as a symbol of disease but also as a reminder of the resilience and creativity that can emerge in times of crisis.

**Figure:**
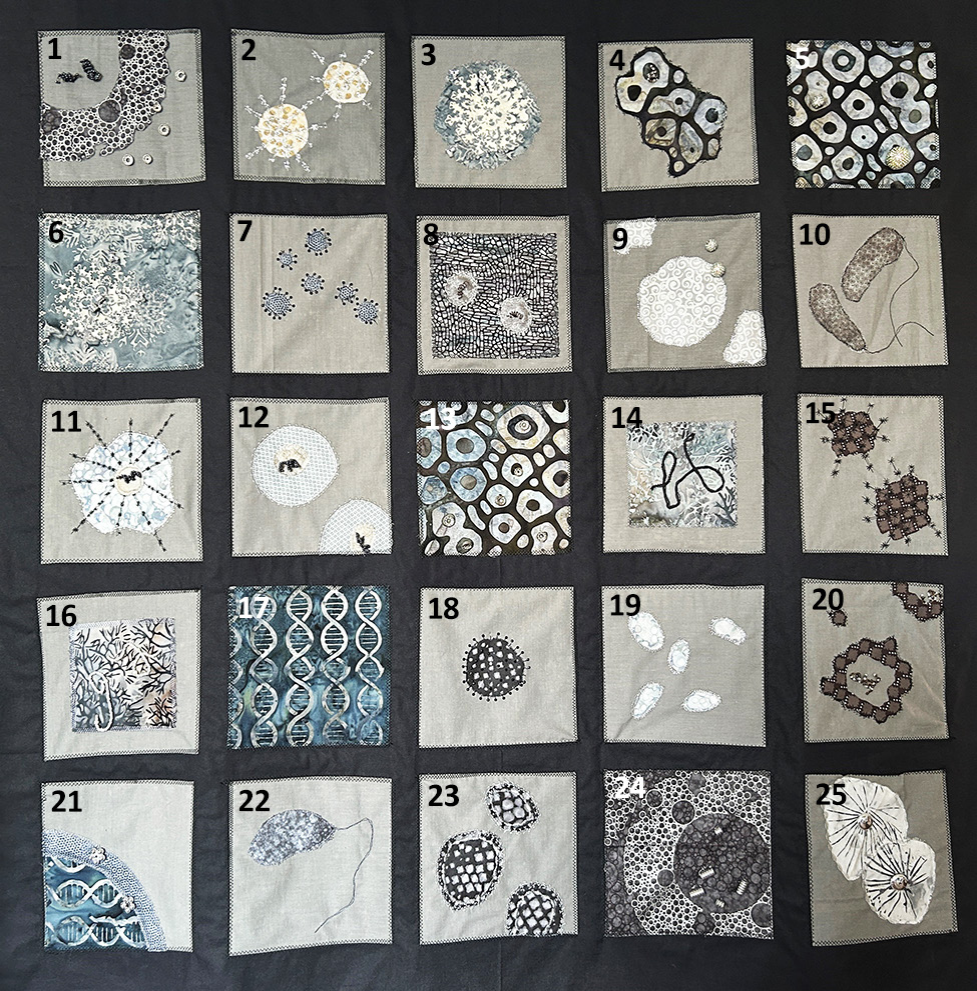
Key for quilt squares in *The Pathogen Quilt*, created by Mija-tesse Ververs, MMed, MPH. Squares 1, 8, 12, and 24, DNA and RNA viruses and replication; squares 2, 3, 6, 12, 18, and 23, pathogens with surface proteins such as glycoproteins, hemagglutinins, or neuraminidases, including HIV, SARS-CoV-2, influenza virus, Nipah virus, and mpox virus; squares 5 and 13, red blood cells; squares 10 and 22, *Vibrio cholerae*; squares 14 and 16, Ebola virus; squares 17 and 21, viral DNA. The remaining squares represent fantasy pathogens, designed for their visual appeal by the artist.

The word quilt is linked to the Latin word culcita, meaning sack or stuffed. Quilting is a method of stitching layers of material together, typically involving 2 layers of fabric with a layer of padding (wadding) in between, held together by lines of stitching based on a pattern or design. Some sources trace the first evidence of quilting back to ancient Egypt, but the history of quilting can be traced at least to medieval times. Quilt-making reflects both the domestic practicality of creating a bedcover through repurposed fabric and a visual demonstration of the design, stitching skills, and cultural traditions of the artist.

In crafting this quilt, Ververs incorporated general features of pathogens, such as viral nucleic acids, as well as the intricate processes of DNA and RNA replication. The building blocks of life—nucleotides—are represented with beads, which also convey a sense of dynamic movement, illustrating particles entering or leaving a cell’s cytoplasm or nucleus or transitioning between pathogen and human tissue. This representation highlights the complex interactions within the microbial world and their impact on human health.

The materials used in this quilt were sourced from the artist’s personal collection during the early months of the COVID-19 pandemic in 2020. The constraint on shopping because of quarantine led to creative choices, including the use of a small pouch from an airline amenity kit. The color palette selected is deliberately muted; Ververs felt that bright, cheerful colors were out of place amidst the somber realities of the time. However, she allowed for the freedom to invent fantasy pathogens, purely for their visual appeal, which became one of the most enjoyable aspects for her of the creative process.

Quilt-making throughout history has combined the practical craft of repurposing fabric with designs and needlework skills that reflect the quilter’s cultural traditions. Ververs’ quilt reflects this rich tradition of quilting as a means of storytelling and is deeply personal to her. Ververs (pers. comm., email, 2025 Aug 12) said:

My mother passed away in late 2019, just before COVID-19. She was an avid quilter, and I had never quilted before. In 2020 and 2021, while working intensively on Ebola Virus Disease and COVID-19 outbreaks, I decided to use some of her fabrics. Quilting became both a way to grieve and a way to feel close to her.

Ververs learned new appliqué techniques while immersed in her work, which focused heavily on nutrition, including breastfeeding and breastmilk, in outbreak contexts. During her 9 years at the Centers for Disease Control and Prevention, her work also focused on diseases such as Ebola virus disease, Marburg virus disease, COVID-19, cholera, dengue fever, Oropouche virus disease, and, more recently, mpox.

The quilt serves a dual purpose: it connects the artist to her mother and provides a creative outlet away from the office. Working on it helped to ground her during the long months of navigating the challenges posed by COVID-19 and brought together her professional and personal passions in a unique way. The intricate designs of *The Pathogen Quilt* invite the viewer’s reflection on the complexities of the microbial world while celebrating the resilience that emerges from shared struggles.
